# Multi-Strain Probiotic Mixture Affects Brain Morphology and Resting State Brain Function in Healthy Subjects: An RCT

**DOI:** 10.3390/cells11182922

**Published:** 2022-09-19

**Authors:** Julia Rode, Hanna M. T. Edebol Carlman, Julia König, Ashley N. Hutchinson, Per Thunberg, Jonas Persson, Robert J. Brummer

**Affiliations:** 1 Nutrition-Gut-Brain Interactions Research Centre, Faculty of Medicine and Health, School of Medical Sciences, Örebro University, 70182 Örebro, Sweden; 2 Department of Radiology and Medical Physics, Faculty of Medicine and Health, Örebro University, 70182 Örebro, Sweden; 3 Center for Lifespan Developmental Research (LEADER), Faculty of Humanities and Social Sciences, School of Law, Psychology and Social Work, Örebro University, 70182 Örebro, Sweden

**Keywords:** resting state functional connectivity, structural changes, brain-derived neurotrophic factor (BDNF), serotonin, mental health, autonomic nervous system, cortisol awakening response, sleep quality, CO_2_ inhalation challenge, gut microbiota

## Abstract

Probiotics can alter brain function via the gut–brain axis. We investigated the effect of a probiotic mixture containing *Bifidobacterium longum*, *Lactobacillus helveticus* and *Lactiplantibacillus plantarum*. In a randomized, placebo-controlled, double-blinded crossover design, 22 healthy subjects (6 m/16 f; 24.2 ± 3.4 years) underwent four-week intervention periods with probiotics and placebo, separated by a four-week washout period. Voxel-based morphometry indicated that the probiotic intervention affected the gray matter volume of a cluster covering the left supramarginal gyrus and superior parietal lobule (*p* < 0.0001), two regions that were also among those with an altered resting state functional connectivity. Probiotic intervention resulted in significant (FDR < 0.05) functional connectivity changes between regions within the default mode, salience, frontoparietal as well as the language network and several regions located outside these networks. Psychological symptoms trended towards improvement after probiotic intervention, i.e., the total score of the Hospital Anxiety and Depression Scale (*p* = 0.056) and its depression sub-score (*p* = 0.093), as well as sleep patterns (*p* = 0.058). The probiotic intervention evoked distinct changes in brain morphology and resting state brain function alongside slight improvements of psycho(bio)logical markers of the gut–brain axis. The combination of those parameters may provide new insights into the modes of action by which gut microbiota can affect gut–brain communication and hence brain function.

## 1. Introduction

Modulation of the gut microbiota by, e.g., probiotics has been shown to affect brain function and behavior (reviewed in [1]). Probiotics are defined as living microorganisms which positively impact the host’s health, if consumed in adequate amounts [2]. Some probiotics may confer a mental health benefit to the host [1]. In preclinical studies, manipulation of the gut microbiota, using probiotic bacteria, has been shown to improve cognition [3,4,5], behavior [3,4,6], stress response [5,7,8], mood [3,4,9,10,11] and physiological parameters [6,10,12,13,14]. In humans, clinical studies indicated that probiotic consumption can impact cognitive function [15,16,17], self-rated subclinical symptoms of affective disorders [11,18,19,20] and potentially reduce stress [15,21], as well as self-reported stress-induced physical and psychological symptoms in healthy subjects [22]. In addition, probiotics have been shown to impact brain activity during specific tasks as well as during an unevoked situation often denoted as resting state [15,16,21,23,24,25,26,27]. Resting state brain function is often used to study the functional connectivity and organization of brain networks (as reviewed in [28]). Intervention for four weeks with a multi-strain probiotic resulted in decreased functional connectivity in a widely distributed network [25]. One functional network that has received considerable attention is the default mode network (DMN) [28]. The DMN, along with the frontal network, has been shown to be downregulated by another multi-strain probiotic intervention, whereas functional connectivity in the salience network (SN) was upregulated [23]. The same study failed to show structural connectivity changes using diffusion tensor imaging. We assessed the effects of a probiotic mixture on resting state brain function in an exploratory way by analyzing some of those networks previously reported to be affected as well as additional ones, side by side with the investigation of probiotic effects on brain morphology using voxel-based morphometry (VBM).

For the first time, we assessed the mental health effect of a combination of *Lactobacillus helveticus* R0052, *Bifidobacterium longum* R0175 and *Lactiplantibacillus plantarum* R1012 (formerly known as *Lactobacillus plantarum*) in humans. This combination has been shown to have positive effects on anxiety- and depressive-like behavior in mice [29]. Additionally, in healthy human subjects, we have previously reported the effects of this probiotic mixture on brain activity and functional connectivity in regions known to regulate emotions and stress response, amongst others [26,27].

The mechanisms by which these probiotics exert their effects in humans are not yet well understood. The lack of mechanistic insight hinders the understanding of the possible probiotic effects on direct brain processing and the involvement of emotional networks in perceived stress and anxiety and the development of potential novel therapies. Therefore, there is a need for studies that elucidate mechanistic pathways and take the bidirectional brain–gut communication into account, including neuropsychological processes of stress, depression and anxiety.

Thus, in the current study, we incorporated a broad spectrum of outcomes with the aim of characterizing the potential modes of action of the intervention effects. The neural effects of the probiotic intervention were investigated by analyzing resting state functional connectivity and autonomic nervous system function, as well as brain morphology. Biomarkers of neuroendocrine gut–brain axis signaling, as well as of gastrointestinal and systemic inflammation, were assessed to elucidate underlying mechanisms of the probiotic effects on mental health. Additionally, the subjects were carefully characterized regarding their mental health, such as depression and anxiety symptoms, perceived stress, sleep quality as well as quality of life.

## 2. Materials and Methods

### 2.1. Design

In this double-blinded, randomized, placebo-controlled crossover study, 22 healthy subjects (age 24.2 ± 3.4 years, 6 male/16 female) ingested a probiotic mixture (in total 3 × 10^9^ colony-forming units (CFU) per day at the end of shelf-life) containing *Lactobacillus helveticus* R0052 (CNCM-I-1722; 2 × 10^9^ CFU), *Lactiplantibacillus plantarum* R1012 (CNCM-I-3736; 8 × 10^8^ CFU) and *Bifidobacterium longum* R0175 (CNCM-I-3470; 7 × 10^7^ CFU) (see Table S1, Supplementary Materials) [26,27]. In short, the participants, who fulfilled the inclusion criteria and did not meet any of the exclusion criteria (see Tables S2 and S3), received the probiotic or a placebo intervention in randomized order for four weeks each, separated by a four-week washout period.

We aimed at investigating the effect of probiotics on mental health in a healthy study population, including the assessment of brain morphology and brain response patterns during rest. Complementary exploratory outcome parameters included psychological health (such as perceived stress, anxiety and depression), quality of life, sleep quality, autonomic nervous system function, markers of importance for microbiome–gut–brain axis interaction and physical activity, amongst others.

The participants were assessed at baseline and subsequently underwent the respective intervention periods. A carbon dioxide (CO_2_) inhalation challenge was performed at baseline. Before and after both of the intervention periods, blood and saliva samples were collected as well as autonomic nervous system measurements were conducted. Questionnaire data were collected throughout the study on a daily or weekly basis. Actigraphy data were collected for one week at baseline and the end of each intervention period. Resting state fMRI was performed after each intervention period. Table S4 presents the key events of the study.

All participants gave their written informed consent prior to participation in the study. The study was approved by the Ethical Review Board of Uppsala, Sweden (registration number: 2017/398 A and B) and registered at ClinicalTrials.gov (NCT03615651). The study was conducted in 2018 according to Good Clinical Practice and in accordance with the Helsinki Declaration of 1975 and its revisions. Any adverse event as well as unintended effect was recorded at each study visit by the study staff [26,27].

The sample size calculation was based on the brain response pattern towards an emotional task and is reported in Rode et al. [27]. In short, considering our primary outcome, we aimed at a power of 80%, a 95% confidence interval (i.e., significance level at 5%), and Bonferroni correction for the multiple testing. Sample size calculation revealed that a minimum of 18 subjects would be required to demonstrate the anticipated difference. A total of 22 subjects were included allowing for a maximum dropout rate of 20%. The randomization, allocation and blinding procedure and participants’ baseline characteristics are reported in Rode et al. and Edebol-Carlman et al. [26,27]. The CONSORT Flow Diagram can be found in the Supplementary Materials.

### 2.2. MRI Protocol

A magnetic resonance imaging (MRI) examination was performed after each intervention period. The imaging protocol included an initial 4.5-min structural scan, followed by a 5-min resting state fMRI (eyes closed). All of the MRI examinations were performed with the same protocol, implying an equal sequential order for all acquisitions. A 3.0T MR system (Discovery 750w, GE Medical Systems, Waukesha, WI, USA) and a 32-channel head coil were used. The structural scan (T1w IR-prepared fast spoiled gradient recalled echo, “BRAVO”) had the following parameters applied; TR/TE = 8.6/3.3 ms, acquired voxel size of 0.9 × 0.9 × 1.2 mm, while the resting state fMRI acquisitions were based on a gradient echo EPI pulse sequence using the following parameters; TR/TE = 2500/35 ms, slice thickness 3.6 mm, no slice gap, in-plane resolution of 3.75 × 3.75 × 3.6 mm and a reduction factor (ASSET) of 2.

The subjects were asked to have the same routines on the day of both MRI examinations, including avoidance of physical exercise and consumption of a maximum of one cup of coffee or tea in the morning.

### 2.3. Autonomic Nervous System Measurement

During rest (sitting, while completing questionnaires, 5 min) before and after each of the intervention periods, autonomic nervous system activity was measured using the BioPac system and software (BioPac Inc., Goleta, CA, USA) as described in detail in Edebol-Carlman et al. [26]. In short, heart rate variability (HRV) as well as sympathetic and vagal activity were assessed.

### 2.4. Biochemical Measurements and Analysis

#### 2.4.1. Saliva Samples Collection and Analysis

Saliva was sampled to investigate cortisol concentrations. For the cortisol awakening response, subjects were instructed to collect saliva samples at home. Saliva samples were collected using Salivette collection tubes (Sarstedt, Nümbrecht, Germany) at five time points on three consecutive days (two weekdays, one day at the weekend) before and after each intervention period, respectively, at time of awakening, as well as 15, 30, 45 and 60 min thereafter. Subjects were instructed not to brush their teeth before completing the saliva sampling to avoid contamination of saliva with blood caused by micro-injuries in the oral cavity. Subjects were asked to refrain from food intake and beverages containing alcohol, caffeine or fruit juices as well as smoking during the sampling period. Besides these restrictions, subjects were free to follow their normal morning routines on the sampling day.

The Salivette collection tubes were kept in the participants’ home refrigerators and transported cooled to the study unit after a maximum of three days. Samples were centrifuged at 1000× *g* for 1 min, transferred to Eppendorf tubes (Eppendorf, Hamburg, Germany), immediately frozen at −20 °C and subsequently stored at −80 °C until analysis. For biochemical analysis, samples were thawed and salivary cortisol levels determined using a chemiluminescence assay with high sensitivity and a minimal detection of 0.44 nmol/L (IBL, Hamburg, Germany) at DresdenLab Service GmbH (Dresden, Germany). Intra- and inter-assay coefficients of variation were below 8%.

#### 2.4.2. Blood Samples Collection and Analysis

Blood samples were collected in the morning after an overnight fast for measurement of brain-derived neurotrophic factor (BDNF), serotonin, intestinal fatty acid-binding protein (I-FABP), and high-sensitivity C-reactive protein (hsCRP) at the same time of the day before and after the probiotic and placebo intervention, respectively. All of the samples were stored for up to six months at −80 °C until subsequent analysis.

The concentration of BDNF in serum (serum separator tube BD Vacutainer^®^ SST^TM^ II Advance (BD, Franklin Lakes, NJ, USA) 8–10 inversions, centrifugation after >30 min, 1000× *g*, 15 min, room temperature) was determined in 1:20 dilutions using the Quantikine ELISA Human Free BDNF Immunoassay (R&D Systems, Minneapolis, MN, USA) according to the manufacturer’s instructions.

The concentration of serotonin in serum (Vacuette Z serum sep clot activator (Greiner bio-one, Kremsmünster, Austria), 8–10 inversions, centrifugation within 30–60 min, 3400× *g*, 10 min, room temperature) was determined using a serotonin ELISA (REF: RE59121; ThermoFisher, Waltman, MA, USA) according to the manufacturer’s instructions at the Clinical Chemistry Laboratory at Sahlgrenska University Hospital (Gothenburg, Sweden).

The concentration of I-FABP in Li-heparin plasma (BD Vacutainer^®^ LH PST^TM^ II (BD), 8–10 inversions, centrifugation within 20 min, 1500× *g*, 10 min, 4 °C, brakes not activated) was determined in 1:2 dilutions using the Human I-FABP ELISA kit (HK406; HycultBiotech, Uden, the Netherlands) according to the manufacturer’s protocol.

The concentration of hsCRP in Li-heparin plasma (BD Vacutainer^®^ LH PST^TM^ II (BD), 8–10 inversions, centrifugation within 30–60 min, 2000× *g*, 7 min, 4 °C, brakes not activated) was determined using the CardioPhase hsCRP assay (REF 06837459; Siemens Healthcare, Erlangen, Germany) using a Siemens ADVIA 1800 Chemistry System (Siemens Healthcare) according to hospital routines.

### 2.5. Questionnaires

The questionnaires assessing general health and psychological symptoms were collected weekly throughout the study. The Euro Qol-Health-related quality of life (EQ-5D-5L) index value is a quantitative measure of health status [30,31]. Subjects rated their current health status between ‘0’ and ‘100’, with ‘100’ being the best health possible. The Hospital Anxiety and Depression Scale (HADS) consists of a subscale for anxiety (seven items) and a subscale for depression (seven items) [32]. Subjects rated each item on a four-point scale with ‘0’ representing the most positive option and ‘3’ the most negative. The ten-item Perceived Stress Scale (PSS) measures the perception of stress to situations in one’s life [33]. Each item was rated on a four-point scale. The results were presented in such a way that a value of ‘4’ indicated a high stress level. The State and Trait Anxiety Inventory for Adults (STAI) is the most commonly used scale for anxiety and is rated on a four-point Likert-scale, with higher values indicating increased anxiety [34]. These questionnaires are commonly used for gut–brain axis studies [11,18,35].

Additionally, participants were asked to complete the Karolinska Sleep Diary (KSD) daily every morning and the Diary of Workload (DOW) daily every evening [36,37,38]. The KSD consists of 16-items concerning the quality of sleep. Those items can be categorized into three subscales: Karolinska Sleepiness Scale (KSS), Sleep Quality Index (SQI) and Awakening Index (AI). The ratings for SQI and AI were made on a five-point scale (‘1’ = poor sleep, ‘5’ = no problems with sleep) and on a nine-point scale for KSS (‘1’ = very alert, ‘9’ = very sleepy, fighting sleep, an effort to stay awake). The DOW is a 16-item self-report instrument for measuring the daily workload in adults. Subjects reported each day with respect to workdays (defined as at least four hours of work during the day), working times (start and finishing), and answered questions about workload at work and outside of work (‘1’ very low—‘5’ very high; for work-free days the workload at work was given a score of ‘0’ for subsequent calculations), physical activity and stress (for both: ‘1’ very low—‘5’ very high), exhaustion, irritation, anxiety and relaxation (for all: ‘5’ not at all—‘1’ very high). In our study, a value of ‘5’ always presented the most negative option. An average DOW score was calculated from all of the questions per day. The DOW also included a question about the perceived health on the day of rating (‘1’ very good—‘7’ very poor). This question was analyzed separately.

### 2.6. Actigraphy

Actigraphs (Actiwatch spectrum-pro; Philips Respironics, Murrysville, PA, USA) were used to assess participants’ physical activity and sleep quality by means of measuring wrist activity for one week at baseline and during the last week of the respective intervention period. Movement data were sampled at a rate of 32 Hz, and activity counts were recorded in 30 s epochs and presented as counts per minute. Four times a day (10 and 13 a.m., 16 and 19 p.m.) the participants scored their current level of stress using an 11 points scale (0 = no stress, 10 = maximal stress). Sleep onset and offset were defined as the first and last periods of 10 min with ≤1 epoch of movement/wake. Sleep quality was assessed as time in bed, total sleep time (absolute and relative), sleep onset latency (minutes between bedtime and initial sleep onset), wake after sleep onset (wake time between initial sleep onset and final sleep offset; absolute and relative) as well as sleep efficiency (total sleep time/time in bed). Recording started and ended at 12 p.m. in order to reduce possible variations due to different morning/evening routines. The data were analyzed using Actiware 6.0 software (Philips Respironics). The automatic detection algorithm was used for initial analysis. Visual inspection of the recordings resulted in the adjustment of three sleep intervals (out of 616 expected sleep intervals). The data were averaged over the course of each recording period prior to statistical analysis.

### 2.7. Analysis of Structural MRI Data

Analysis of the structural MRI data was performed using SPM12 (Statistical Parametric Mapping, The Wellcome Centre for Human Neuroimaging, UCL Queen Square Institute of Neurology, London, UK) in MATLAB R2020b (The Mathworks Inc., Natick, MA, USA) using default settings for voxel-based morphometry (VBM) if not stated otherwise. The BRAVO scans were segmented choosing native and Dartel-imported segmentation for gray and white matter. Then, a Dartel template was created, the images normalized to Montreal Neurological Institute (MNI) space and smoothed with a Gaussian kernel set to 8 mm. A one-sample *t*-test was designed using one delta image (probiotics–placebo) per subject. Total intracranial volume was included as covariate. No implicit or explicit masking was applied. The cluster-defining threshold was set to *p* < 0.0001. Finally, an F-contrast examining structural differences in gray matter between probiotics and placebo was assessed and suprathreshold results reported. To test for directionality, additionally, two T-contrasts were examined (probiotics > placebo and placebo > probiotics). The MRI results were visualized using MRICroGL (version 1.2.20210317) [39].

### 2.8. Analysis of Resting State fMRI Data

The analysis of resting state fMRI was performed in CONN functional connectivity toolbox version 18.b standalone [40] using MATLAB 9.5 R2018b (The Mathworks Inc.). The CONN default preprocessing pipeline was used for data preprocessing with conservative settings (95th percentile) for ART-based outlier detection for scrubbing. Functional and structural scans were realigned, slice-time corrected, segmented and normalized into MNI-space before smoothing using a Gaussian kernel (8 mm). Denoising was performed with sequential regression (RegBP), a band-pass filter of 0.008 to 0.09 Hz, linear detrending and no despiking. The data of two subjects were excluded due to data-quality constraints. The interventions were compared with respect to functional connectivity. A seed-to-voxel analysis (bivariate correlation, HRF weighting) with seed regions within eight networks (default mode network, salience network, frontoparietal network, language network, cerebellar network, dorsal attention network, sensorimotor network, visual network) was performed. A two-sided, parametric test was performed, voxel threshold was set to *p* < 0.001, uncorrected, and cluster threshold was set to *p* < 0.05 false discovery rate (FDR)-corrected. Brain regions were annotated using CONN and Harvard-Oxford atlas. The software multi-image analysis GUI (MANGO, Research Imaging Institute, University of Texas Health Science Center, San Antonio, TX, USA) was used to view functional images.

### 2.9. Statistical Analysis of Other Markers

The data of the blood markers, autonomic nervous system activity, parameters of sleep and stress ratings by Actigraphy were tested for normal distribution using the Shapiro–Wilk test. The baseline-corrected effects of the probiotic and the placebo intervention, respectively, were analyzed using paired samples *t*-tests for normally distributed data (serotonin, I-FABP, vagal activity and sympathetic activity, absolute sleep time, absolute wake time after sleep onset, sleep onset latency and sleep efficiency, extreme stress) or Wilcoxon matched-pairs signed rank for non-normally distributed data (BDNF, hsCRP and HRV, relative sleep time and wake time after sleep onset and average stress ratings by Actigraphy) using GraphPad Prism version 8 (GraphPad Software Inc.; USA). Median and interquartile range (IQR 25 to 75) are presented for all of those markers, as well as for the ones that were normally distributed. For the analysis of hsCRP, the results of one subject were excluded due to the concentration before the placebo intervention being seven standard deviations higher than the mean of all samples.

Additionally, a one-sample *t*-test (serotonin, I-FABP) and a one-sample Wilcoxon test (BDNF, hsCRP) were performed to analyze if baseline-corrected values differed from zero (GraphPad Prism version 8).

The questionnaires were analyzed using repeated measures ANOVAs of baseline-corrected scores with respect to treatment, time (weekly measures) and treatment–time interaction effects on original scale using SPSS Statistics version 26 (IBM Corp., North Castle, NY, USA). Residuals of questionnaire analyses were checked for approximate normality by visual inspection (see the Supplementary Materials). The daily questionnaire ratings (KSD and DOW) were averaged per week prior to ANOVA. Post hoc multiple comparisons were performed with Sidak’s correction. Mean ± standard deviation is presented for all of the questionnaire data.

For the cortisol awakening response, the results of each time point of the morning salivary cortisol profiles were averaged over the three days measured. Of those, the area under the curve (AUC) as well as the total average of all five samples was calculated [41]. Normal distribution was assessed by the Shapiro–Wilk test. As the data were non-normally distributed, baseline-corrected effects of probiotic and placebo intervention, respectively, were analyzed using a Wilcoxon matched-pairs signed rank test using GraphPad Prism version 8.

Generally, to allow for hypothesis generation and to reveal potential modes of action, *p* > 0.05 are also reported.

## 3. Results

### 3.1. Anatomical Structure of Individual Brain Regions was Significantly Affected by Probiotic Intervention

**Figure 1:**
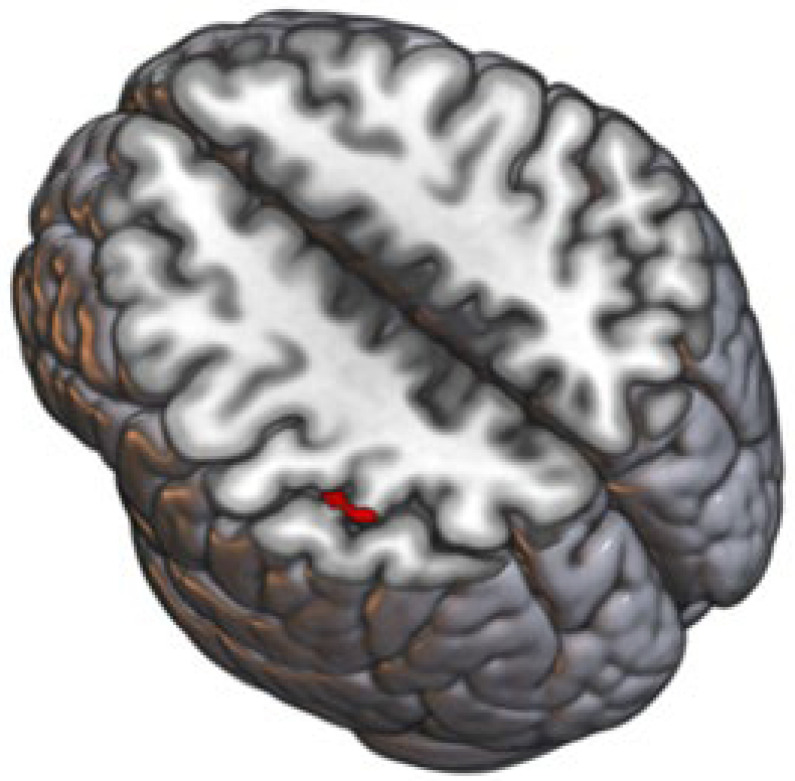
Effect of probiotic intervention on the anatomical structure of the brain. Gray matter of a cluster covering left supramarginal gyrus and superior parietal lobule was significantly (*p* < 0.0001) altered upon probiotic intervention. Schematic visualization of the affected brain region (red).

VBM indicated that the gray matter volume of a cluster with peak coordinates −45 –39 52 (x y z, based on the Montreal Neurological Institute (MNI) space) covering the left supramarginal gyrus and superior parietal lobule was significantly altered upon probiotic intervention (k = 36, F = 34.63) (Figure 1). Those alterations were driven by a decrease in gray matter volume upon probiotic intervention.

### 3.2. Functional Brain Connectivity during Rest Was Significantly Affected by Probiotic Intervention

The functional connectivity of brain regions within known networks with the rest of the brain was significantly altered comparing the probiotic intervention to the placebo (Table 1).

In detail, the probiotic intervention resulted in significantly increased functional connectivity of the default mode network (DMN) with the postcentral gyrus and the superior parietal lobule (FDR = 0.027). Furthermore, increased functional connectivity between the language network and several brain regions including the middle temporal gyrus, the inferior temporal gyrus and the lateral occipital cortex were observed after the probiotic intervention compared to the placebo (FDR = 0.049).

The probiotic intervention resulted in significantly reduced functional connectivity of the left supramarginal gyrus (within the salience network (SN)) with the postcentral gyrus (FDR = 0.043) and of the right supramarginal gyrus (within SN) with the brain stem, precuneus cortex, intracalcarine cortex, cerebellum and supracalcarine cortex (FDR = 0.034 and 0.046). Finally, reduced functional connectivity was also observed between the frontoparietal network and the middle frontal and precentral gyri after the probiotic intervention compared to the placebo (FDR = 0.029).

Functional connectivity between seed regions within the cerebellar network, dorsal attention network, sensorimotor network and visual network and any other region of the brain was not affected by the probiotic intervention.

### 3.3. Autonomic Nervous System Activity during Rest Was Not Significantly Affected by the Probiotic Intervention

**Figure 2:**
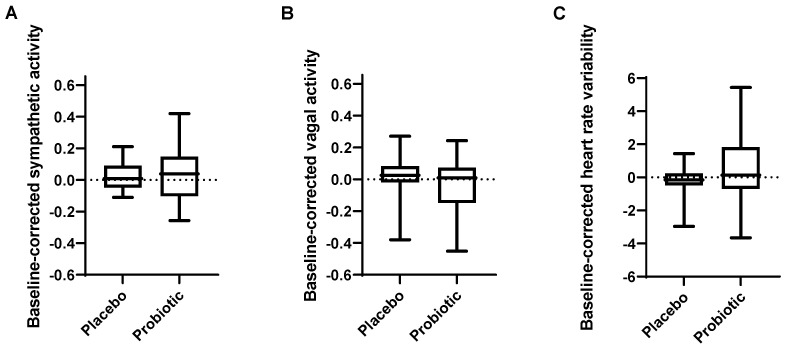
Effect of probiotic intervention on autonomic nervous system function. Baseline-corrected (**A**) sympathetic activity, (**B**) vagal activity, (**C**) heart rate variability. Line presents median, box presents 25th and 75th percentile, whiskers present minimum and maximum.

Baseline-corrected sympathetic and vagal activity during rest were not affected by probiotic intake (*p* = 0.758 and *p* = 0.164, respectively) (Figure 2A,B). Similarly, baseline-corrected heart rate variability (HRV) was not affected (*p* = 0.276, Figure 2C).

### 3.4. Blood Markers Gave Insights into Possible Modes of Action

#### 3.4.1. Probiotic Intervention Did Not Affect Systemic or Gastrointestinal Inflammation

**Figure 3:**
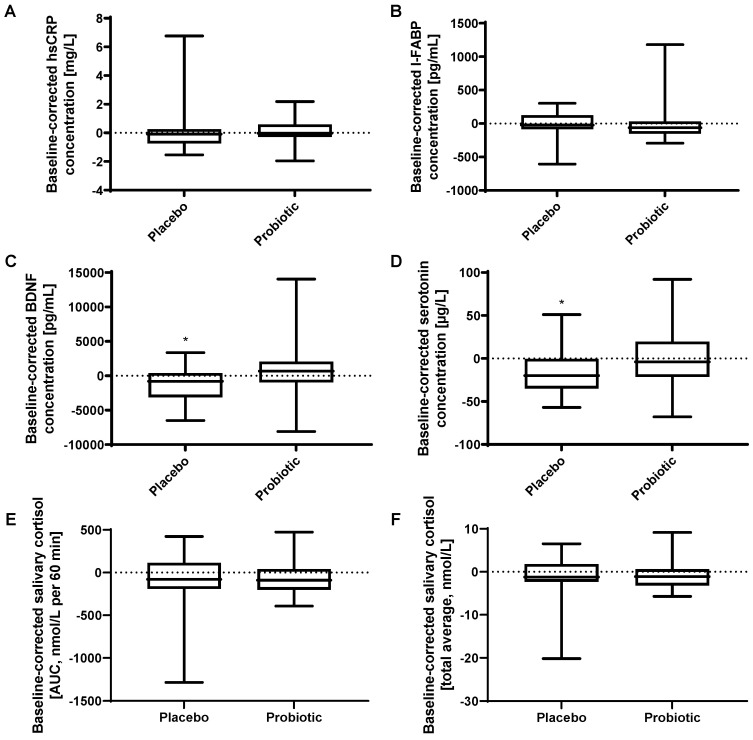
Effect of probiotic intervention on biomarkers. Baseline-corrected concentration of (**A**) high-sensitivity C-reactive protein in plasma, (**B**) intestinal fatty acid-binding protein in plasma, (**C**) brain-derived neurotrophic factor in serum, (**D**) serotonin in serum. Salivary cortisol awakening response calculated as (**E**) area under the curve and (**F**) total average. Line presents median, box presents 25th and 75th percentile, whiskers present minimum and maximum, * *p* < 0.05 for the comparison before vs. after intervention.

High-sensitivity C-reactive protein (hsCRP) (*p* = 0.740) and intestinal fatty acid-binding-protein (I-FABP) (*p* = 0.633) in plasma were not significantly affected by the probiotic intervention (Figure 3A,B).

#### 3.4.2. Probiotic Intervention and Neuroendocrine Signaling

Brain-derived neurotrophic factor (BDNF) in serum was not significantly (*p* = 0.121) affected by the probiotic intervention (Figure 3C). Serotonin in serum was not significantly (*p* = 0.081) affected, but showed a trend towards a higher baseline-corrected concentration after probiotic intervention (median of −4.0 µg/L (IQR −21.5 to 19.5)) compared to after placebo (median of −20.0 µg/L (IQR −35.0 to −0.5)) (Figure 3D). Both BDNF as well as serotonin concentrations decreased significantly during the placebo intervention period (BDNF: *p* = 0.033; serotonin: *p* = 0.014), but not during the probiotic intervention period (BDNF: *p* = 0.388; serotonin: *p* = 0.986).

### 3.5. Cortisol Awakening Response Was Not Significantly Affected by Probiotic Intervention

The cortisol awakening response was assessed by repetitive salivary cortisol measurements. No differences were detected between the interventions with a baseline-corrected median AUC of −89.5 nmol∗L∗h (IQR −200.1 to 41.6) after the probiotic intervention compared to −79.0 nmol∗L∗h (IQR −190.3 to 114.4) after the placebo (*p* = 0.633) (Figure 3E). Measures of baseline-corrected total averages of morning salivary cortisol concentrations showed similar results with a median of −1.1 nmol/L (IQR −3.2 to 0.6) after probiotic and −1.2 nmol/L (IQR −2.3 to 1.8) after placebo intervention (*p* = 0.610) (Figure 3F).

### 3.6. Effect of Probiotic Intake on Psychological Symptoms and General Health

**Figure 4:**
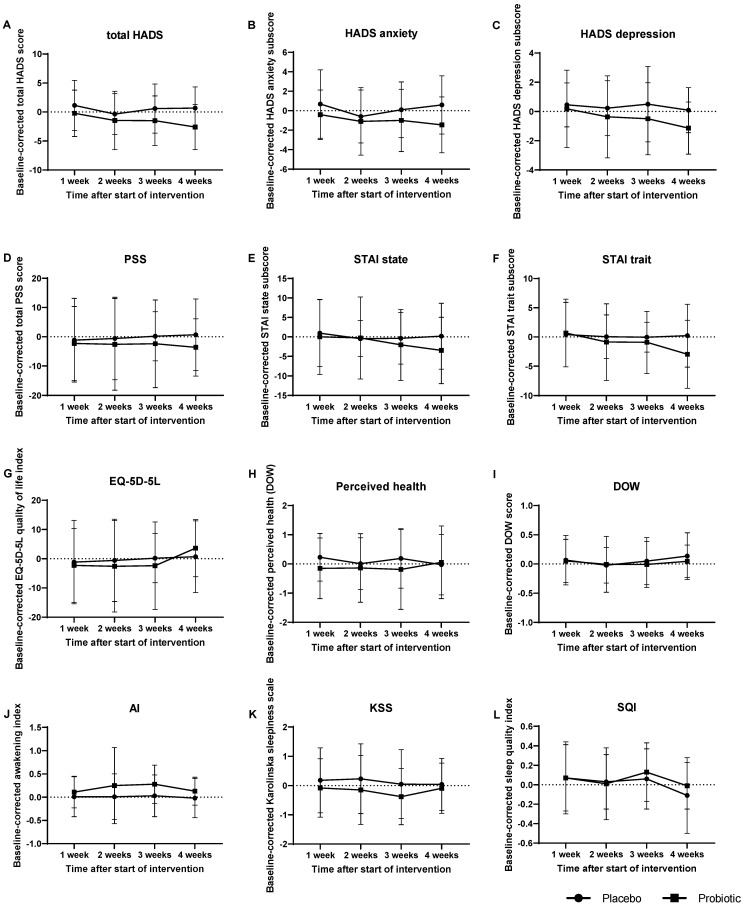
Effect of probiotic intervention on psychological symptoms, general health and sleep quality. (**A**–**C**) HADS—Hospital Anxiety and Depression Scale; (**D**) PSS—Perceived Stress Scale; (**E**,**F**) STAI—State and Trait Anxiety Inventory; (**G**) EQ-5D-5L—Euro Quality of Life version 5D-5L; (**H**) Perceived health (subscale of DOW); (**I**) DOW—Diary of Workload; (**J**) AI—Awakening Index; (**K**) KSS—Karolinska Sleepiness Scale; (**L**) SQI—Sleep Quality Index. Baseline-corrected; mean and standard deviation are presented.

**Figure 5:**
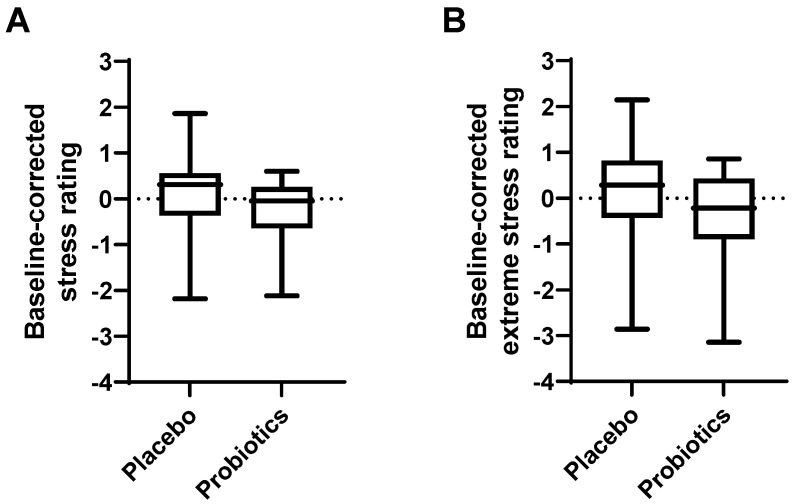
Effect of probiotic intervention on Actigraphy stress measurements. (**A**) Baseline-corrected average of daily stress ratings and (**B**) extreme daily stress ratings. Line presents median, box presents 25th and 75th percentile, whiskers present minimum and maximum.

Psychological symptoms and general health were assessed by questionnaires. The effects of the intervention on self-rated stress, depression and anxiety scores, perceived health and quality of life are shown in Table 2 and Figure 4. Hospital Anxiety and Depression Scale (HADS) scores were decreased by the probiotic intervention compared to the placebo (total HADS *p* = 0.056, anxiety sub-score *p* = 0.153, depression sub-score *p* = 0.093), however, this did not reach significance. Post hoc analysis revealed that for both the total HADS and the depression sub-score these differences were derived from differences at week four. Baseline-corrected average scores of total HADS at week four were −2.6 ± 3.9 after probiotic intervention compared to 0.7 ± 3.6 after placebo (*p* = 0.005). Baseline-corrected average HADS depression sub-scores at week four were −1.1 ± 1.8 after probiotic intervention compared to 0.1 ± 1.5 after placebo (*p* = 0.019). For the depression sub-score of HADS, a repeated measures ANOVA also yielded significant results for the effect of time (*p* = 0.027) independent of the interventions. The State and Trait subscale (STAI) scores, Perceived Stress Scale (PSS), average and extreme stress ratings (by Actigraphy, Figure 5) as well as the perceived health and perception of daily workload (both assessed by DOW) were not significantly affected by the interventions. A two-way repeated measures ANOVA of the index variable of quality of life (assessed by EQ-5D-5L) yielded no significant effect of treatment (*p* = 0.804), but for time (*p* = 0.029).

### 3.7. Sleep Quality was slightly Affected by Probiotic Intervention

**Figure 6:**
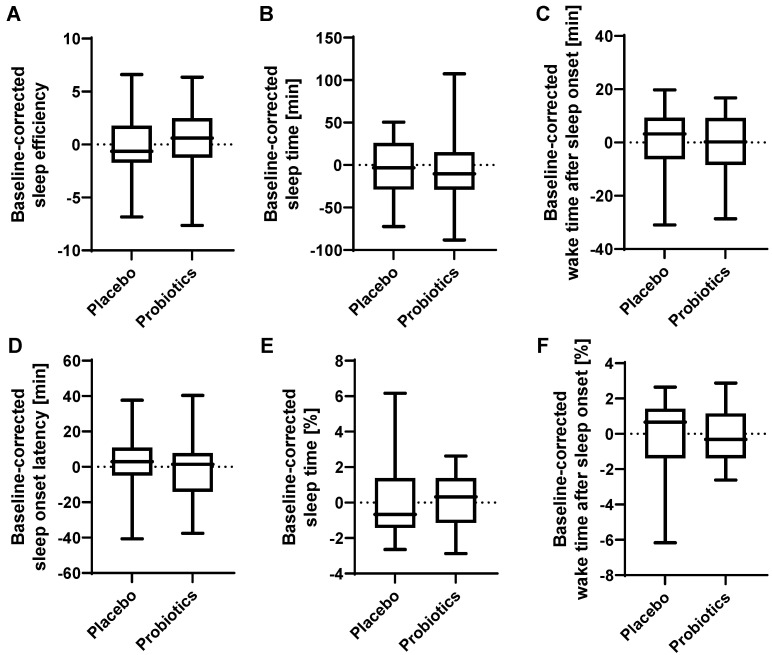
Effect of probiotic intervention on Actigraphy sleep measurements. Baseline-corrected (**A**) sleep efficiency (total sleep time/time in bed), (**B**) absolute sleep time, (**C**) absolute wake time after sleep onset, (**D**) sleep onset latency, (**E**) relative sleep time, (**F**) relative wake time after sleep onset. Line presents median, box presents 25th and 75th percentile, whiskers present minimum and maximum.

To assess the overall sleep quality, several parameters were assessed. The ratings on the Awakening Index were slightly, albeit non-significantly, increased after the probiotic intervention compared to placebo (*p* = 0.058), indicating a subtle improvement in sleep patterns (Table 2 and Figure 4J). Post hoc analysis revealed no significant differences in the Awakening Index at the individual time points. The ratings on the Karolinska Sleepiness Scale were not significantly altered upon probiotic intervention (*p* = 0.299, Figure 4K). The ratings on the Sleep Quality Index (Figure 4L) were not significantly affected by probiotic intervention (*p* = 0.727), but by time (*p* = 0.031) independent of the interventions. Absolute and relative sleep time, absolute and relative wake time after sleep onset, sleep onset latency and sleep efficiency were not affected by the probiotic intervention (assessed by Actigraphy complementary to daily diaries) (Figure 6).

## 4. Discussion

Gut microbiota modifications, by probiotics amongst others, were shown to potentially affect brain activity and behavior via the gut–brain axis. In this study, we evaluated the novel concept that probiotic intervention may affect brain morphology. Indeed, we showed that a four-week intervention with a probiotic product containing *Bifidobacterium longum* R0175, *Lactobacillus helveticus* R0052 and *Lactiplantibacillus plantarum* R1012 altered gray matter volume and the brain response patterns at rest in identical or proximal brain regions. Furthermore, the probiotic intervention had subtle effects on psychological health, such as depression ratings and sleep patterns, as well as markers of gut–brain interactions, such as serum serotonin concentrations. All of those effects occurred without substantial effects on the gut microbiota composition, as reported previously [27]. This report is one of the few human probiotic intervention studies investigating effects on the gut–brain axis in more detail and elucidating potential modes of action.

Interestingly, the changes seen in resting state connectivity and gray matter structure seemed to be specific to a few networks and brain regions as connectivity and brain structure were not just generally altered.

The interpretation of the short-term changes in brain structure, such as gray matter volume, remains challenging [42]. Previous studies have shown that the structure of the brain, predominantly measured as gray matter volume, is associated with gut microbiota composition and its diversity in healthy adults [43,44,45]. Gray matter alterations have been associated with a variety of diseases, and thus intervention-related changes might give hints on modes of action of gut microbiota (modifications) on brain function as well as possible treatment options.

The probiotic intervention resulted in gray matter volume changes in a cluster spanning over the supramarginal gyrus and superior parietal lobule in the left hemisphere closely neighboring the postcentral gyrus. These are regions that are also among those with altered resting state functional connectivity. The functional connectivity between the left supramarginal gyrus and the left postcentral gyrus decreased significantly upon probiotic intervention. In addition, the right supramarginal gyrus showed decreased functional connectivity with two clusters. The supramarginal gyrus is part of the salience network, a behavioral relevant network with involvement in emotional processing, amongst others. It is notable that no other region of the salience network (anterior cingulate cortex, anterior insula left/right, rostral prefrontal cortex left/right) showed altered functional connectivity with any cluster. Moreover, a cluster covering the superior parietal lobule and postcentral gyrus, although located in the right hemisphere, showed increased functional connectivity with the predominant resting state network, the default mode network, upon probiotic intervention.

Reduced gray matter volume alongside reduced functional connectivity may indicate higher brain efficiency. The fact that these changes were observed in brain regions implicated in emotional regulation corresponds well with improved mood as indicated by the weekly questionnaire ratings. Furthermore, these results are consistent with our previously reported study in which we observed dampened reactivity towards acute negative emotional challenges upon an identical probiotic intervention [27].

We have also previously reported that our probiotic intervention can alter brain response patterns to a task with an acute multi-target stressor [26]. Resting state fMRI presents a less resource demanding approach to measure brain function, but has not yet been used frequently in the context of probiotic intervention studies. Our findings support that even a mild four-week intervention, such as the intake of probiotics, is sufficient to evoke changes in otherwise robust brain functions during an unevoked situation, thus indicating probiotic effects in the absence of additional acute mental challenges. This approach has so far only been employed by two other studies with different probiotic mixtures [23,25]. Few studies have assessed the effect of supplementation with other probiotic mixtures on resting state brain function using the lower-resolution method electroencephalography [15,21,46].

The application of tasks in the fMRI environment is much more demanding than the application of a resting state scan. Thus, the presented results together with our previously reported ones [26,27] are an important step in order to determine if resting state fMRI could be used as a surrogate marker of mental health effects to facilitate probiotics’ research, or in broader terms nutritional studies, in larger groups.

The reported changes in brain morphology and functional connectivity could be a result of intervention-related changes in sleep patterns, BDNF, serotonin or depression, as revealed by a number of exploratory outcomes including general and psychological health and markers of unevoked gut–brain axis signaling. The assessment of many of these parameters aimed to provide (mechanistic) insights into possible modes of action.

The main bidirectional route of communication between the gut and the brain is the autonomic nervous system. To the best of our knowledge, this is the first study which investigated the effects of probiotics on the autonomic nervous system function at rest. Our combination of probiotic strains did not affect the autonomic nervous system function at rest in the present study, nor during a stressful situation as we reported previously [26]. Similarly, no effect of a two-week multi-strain probiotic intervention on cardiovascular function at rest or during recovery from stress was reported previously [47]. These results suggested that fMRI of resting state may be more sensitive to detect probiotic effects on mental function than the assessment of autonomic nervous system function.

Probiotic effects could be initiated at the side of the gut and exert their effects systemically. Our results suggest that the probiotic intervention did not trigger a sustained mucosal nor a systemic immune response. None of the blood markers were significantly altered by the intervention. The hsCRP and the I-FABP levels measured in the current study were in the range of normal reference values for healthy, young subjects. As the hsCRP and I-FABP concentrations were rather low at baseline, it was difficult to expect a further reduction due to the intervention.

A possible mechanism by which probiotics might exert their effects on brain function is via signaling molecules, such as BDNF and serotonin, which are part of a major route of communication between the gut, its microbiota-rich intraluminal ecosystem, as well as its enteric nervous system and the brain. Gut microbiota depletion has been associated with reduced levels of BDNF and serotonin in preclinical studies and negative behavioral changes and stress in animal models and humans [48,49,50,51,52,53,54]. Whereas the BDNF concentrations did not differ significantly between probiotic intervention and placebo, baseline-corrected serotonin concentrations tended to be higher after the probiotic intervention compared to the placebo, however, this did not reach significance. Interestingly, this seemed to be a result of significantly decreased serum serotonin concentrations after the placebo intervention, but not after the probiotic intervention. Preclinical studies reported that administration of the same probiotic species, albeit not identical strains as included in our study, increased levels of hippocampal BDNF and serotonin in the hippocampus and frontal cortex in rats and mice in addition to reducing anxiety- and depressive-like behavior [3,50,55,56,57]. Even if the effects in our study were subtle, they might provide mechanistic insights into how the gut–brain signaling is affected by the administration of the probiotic product in humans.

Probiotic effects on psychological symptoms have been reported previously. However, the literature about potential psychological effects of probiotics of the species *Bifidobacterium longum*, *Lactobacillus helveticus* and *Lactiplantibacillus plantarum* is equivocal, with some studies reporting effects and others reporting none [11,15,18,35,58]. Yet, probiotic interventions with the three species contained in the probiotic product of the present study have been shown to decrease depression and anxiety scores in healthy study populations (using HADS and the Depression, Anxiety and Stress Scale (DASS)) [11,18,35]. Most previous reports of the stress-reducing effects of the probiotics administered in this study were based on the stress sub-scores of DASS which covers a broader spectrum of items than the PSS [35]. Nevertheless, a few reports also showed a reduction in perceived stress assessed by PSS [15].

In the present study, however, none of the questionnaires nor the Actigraphy stress ratings were associated with significant changes due to the intervention, even if HADS scores tended to be decreased by the probiotic intervention compared to the placebo. Although none of these scores improved significantly upon probiotic intervention, the absolute decrease of HADS scores with about −3 on total HADS and about −1 to −2 on the sub-scores was remarkable with respect to the fact that baseline values did not indicate any presence of symptoms of affective disorders. The overall mean baseline scores of the HADS, the STAI, the PSS and the Quality of Life (EQ-5D-5L) were as expected in healthy participants. Again, similar to hsCRP and I-FABP, this explains why it was difficult to detect further improvements due to the probiotic intervention. Nevertheless, the CO_2_ inhalation challenge (Supplementary Materials) confirmed that the greater majority of participants included in this study were susceptible to anxiety symptoms.

Apart from assessing perceived stress using questionnaires, we also measured the subjects’ stress levels by salivary cortisol in order to investigate another major communication route between the gut and the brain, namely the hypothalamic–pituitary–adrenal axis. In accordance with the subjective stress levels (assessed using PSS), the cortisol awakening response was not affected by the probiotic intervention in this study population comprising healthy subjects. Similarly, we have previously reported that cortisol levels, as a response to a stressful condition, were not significantly affected by the probiotic intervention [26]. A study by Messaudi et al. in which healthy adults consumed *Lactobacillus helveticus* and *Bifidobacterium longum* for 30 days suggested decreased urinary cortisol levels in the probiotic intervention group compared to baseline, but did not provide statistical proof that cortisol levels differed compared to placebo [18]. Even in stressed adults, a probiotic intervention of *Lactiplantibacillus plantarum* P8 over twelve weeks did not significantly decrease plasma cortisol levels [35].

Studies using other *Lactobacilli* reported decreased salivary cortisol secretion [8] as well as no effect [59] in healthy subjects during a period of increased stress. Interestingly, one study administering prebiotics reported a lower salivary cortisol awakening response in healthy subjects after a three-week intervention [60]. The inconclusiveness regarding probiotic effects on cortisol secretion might derive from different daily life stress of the study populations and from strain specificity, amongst others.

Although our study population did not seem to be particularly stressed, a subtle, albeit non-significant, effect on sleep patterns was seen upon probiotic intervention. The probiotic intervention solely affected subjective sleep ratings and not objective measurements by Actigraphy. This might be an indication that participants experienced a qualitatively better sleep without changes in actual sleep times upon probiotic intervention. These results are in line with previous studies where the intake of specific *Lactobacilli* strains has been reported to help maintain sleep quality during periods of increased stress [59,61]. However, one study also found no effect of a probiotic intervention on sleep quality in non-stressed, healthy adults [46]. This is especially interesting since sleep quality could act as a possible predictor of mood disturbances or their improvement.

Limitations of this study include a rather small sample size. Notably, this study assessed the effects of the probiotic mixture on a study population of men and women, although sex differences might influence outcome parameters. The small sample size, however, hampered subgroup analyses. Furthermore, the choice of a healthy study population might have prevented detection of small improvements of values that were already within a physiological normal range at baseline. Another limitation might be potential seasonal effects. However, the crossover design, with its ability to compensate for such effects, strengthened this study. It is notable that the present study included a wide variety of outcome parameters in order to elucidate potential modes of action. However, this selection was not exhaustive. Nevertheless, the absence of large effects should not be interpreted as a failure, but as promising for subjects with health issues such as psychiatric disorders because we confirmed that a mild intervention evoked positive effects even in a healthy population.

## 5. Conclusions

The study results indicated that changes in baseline brain morphometry and functional brain connectivity are possible without clear or large changes in mood/anxiety, perceived stress as well as general biomarkers of gut–brain axis signaling. Hence, resting state functional magnetic resonance imaging might be a sufficient surrogate marker for the assessment of mental health in the context of probiotic, or in broader terms, nutritional studies. The shown intervention-dependent changes in gray matter strengthen the hypothesis of potential probiotic effects on brain morphology, even in healthy subjects, and contribute to, in conjunction with brain connectivity changes, a greater understanding of the potential modes of action by which probiotics exert their effects via bidirectional gut–brain communication. Even if the effects were subtle, the results also indicated more generally that the specific probiotic strains may have the potential to affect mental health.

## Figures and Tables

**Table 1:** Resting state functional connectivity. Clusters that were found to be associated with significant changes in functional connectivity between both of the interventions during resting state. All other seed regions within the default mode network, salience network, dorsal attention network, frontoparietal network, cerebellar network, sensorimotor network, visual network and language network did not show significantly different connectivity with any other part of the brain. Upward arrows indicate increased and downward arrows indicate decreased resting state functional connectivity after the probiotic compared to the placebo intervention. (Seed-to-voxel analysis).

Seed Region	Coordinates of Peak of the Cluster (x y z)	Cluster Size (mm^3^)	Anatomical Region	T (Probiotic− Placebo)	FDR	Probiotic− Placebo
Default mode network— Medial Prefrontal Cortex	+32 –34 +62	1776	Postcentral Gyrus right, Superior Parietal Lobule right	6.08	0.027	↑
Salience network— left Supramarginal Gyrus	−62 −16 +38	1784	Postcentral Gyrus left	−6.03	0.043	↓
Salience network—right Supramarginal Gyrus	+06 −66 +14	1744	Precuneus cortex, Intracalcarine cortex right and left, supracalcarine cortex right and left	−5.06	0.034	↓
	+06 −34 −40	1336	Brain stem, cerebellum 9 right	−5.68	0.046	↓
Frontoparietal network— left Posterior Parietal Cortex	−38 +12 +38	1816	Middle Frontal Gyrus left, Precentral Gyrus left	−6.65	0.029	↓
Language network— right Inferior Frontal Gyrus	+54 −56 –04th	1104	Middle Temporal Gyrus (temporooccipital part right), Inferior Temporal Gyrus (temporooccipital part right), Lateral Occipital Cortex (inferior division right)	5.73	0.049	↑

**Table 2:** Effect of treatment and time on psychological symptoms, general health and sleep quality. Results from the repeated measures ANOVA of the Hospital Anxiety and Depression Scale (HADS); the Perceived Stress Scale (PSS); the State and Trait Anxiety Inventory for adults (STAI); the Euro Quality of Life 5D-5L Index variable (EQ-5D-5L); perceived health (DOW) and perception of daily workload (DOW); Karolinska Sleep Diary’s (KSD’s) Awakening Index (AI), Karolinska Sleepiness Scale (KSS) and Sleep Quality Index (SQI).

Questionnaire	Subscale	Treatment Effect (*p*-Value)	Time Effect (*p*-Value)	Treatment-Time Interaction Effect (*p*-Value)
HADS	Total score	0.056	0.175	0.519
HADS	Anxiety score	0.153	0.304	0.469
HADS	Depression score	0.093	0.027	0.676
PSS	Total score	0.136	0.303	0.669
STAI	State score	0.427	0.223	0.455
STAI	Trait score	0.369	0.364	0.538
EQ-5D-5L	Quality of life index score	0.804	0.029	0.286
DOW	Perceived health	0.478	0.848	0.462
DOW	Perception of workload	0.736	0.179	0.816
KSD	AI	0.058	0.537	0.672
KSD	KSS	0.299	0.549	0.471
KSD	SQI	0.727	0.031	0.658

## Data Availability

Underlying deidentified data will be made available upon request pending ethical approval and a signed agreement.

## Supplementary Materials

The following supporting information can be downloaded at: https://www.mdpi.com/article/10.3390/cells11182922/s1, CONSORT Flow Diagram; Table S1: Composition of the probiotic product; Table S2: Inclusion criteria; Table S3: Exclusion criteria; Table S4: Schedule of study events; Residuals for questionnaire analysis—normality check; CO_2_ challenge test (Method, Results, Table S5). References [62,63] are cited in Supplementary materials.
