# Supplementary material for: Multi-Strain Probiotic Mixture Affects Brain Morphology and Resting State Brain Function in Healthy Subjects: An RCT
Source: Cells. 2022 Sep 19;11(18):2922. doi: 10.3390/cells11182922 (PMC9496704; doi:10.3390/cells11182922)
Supplement: Supplementary file 1 [file cells-11-02922-s001.zip › Supplement_PROBIO_VBM+REST.pdf]

SUPPLEMENTARY INFORMATION

Multi-strain probiotic mixture affects brain morphology and resting state brain function in healthy subjects: An RCT

Julia Rode, Hanna MT Edebol Carlman, Julia König, Ashley N Hutchinson, Per Thunberg, Jonas Persson, Robert JM Brummer.

Supplementary Table S1. Composition of the probiotic product

**Active Ingredients**	**Amount per sachet**
**Inulin**	500 mg
**Magnesium**	45 mg
**Potassium**	50 mg
**Zinc**	9 mg
**Glutathione**	20 mg
**Lactoferrin**	10 mg
**Probiotic blend: *L. helveticus R0052 (CNCM-I-1722), B. longum R0175 (CNCM-I-3470), L. plantarum R1012 (CNCM-I-3736)***	3 x 10^9 CFU

Ingredients:
Fructose, Inulin, Magnesium Gluconate; Acidifying agent: Citric Acid; Aroma, Potassium Citrate, Mixture of live lactic cultures (*Lactobacillus Acidophilus* (*Helveticus*), *Bifidobacterium Longum*, *Lactiplantibacillus Plantarum*), Zinc Gluconate, Magnesium Oxide; Anti-caking agent: Silicon Dioxide; Glutathione; Sweeteners: Acesulfame K, Sucralose; Maltodextrin, Lactoferrin; Dyes: E102, E124. (underlined ingredients were also contained in the placebo)

Supplementary Table S2. Inclusion criteria

1. 18-65 years of age
2. Signed informed consent

Supplementary Table S3. Exclusion criteria

3. Concurrent or recent (< twelve weeks) treatment with drugs affecting intestinal function or mood, e.g. antidepressants or antibiotics
4. Concurrent or recent (< four weeks) use of nutritional supplements or herb products affecting intestinal function or mood (e.g. aloe vera, St. John’s Wort, fibres, prebiotics and probiotics)
5. Diagnosis of major psychiatric or somatic disease
6. Abuse of alcohol or drugs
7. Recent (< four weeks) intake of proton pump inhibitors (e.g. omeprazol)
8. Asthma
9. Cardiovascular diseases
10. Epilepsy
11. Renal failure
12. Cerebral bleeding or history of cerebral bleeding
13. Allergy to latex
14. Pregnancy (assessed by urine test) or breastfeeding
15. Claustrophobia
16. Smoking or using tobacco including snuff

17. Inability to maintain exercise routine and dietary pattern during the study
18. Consumption of more than six cups of coffee/caffeine-containing beverages per day
19. Professional athlete
20. Any contraindication to an MRI (e.g. medical implant or device not compliant with MRI)
21. Recent (< three months) regular intake of systemic corticosteroids and anti-inflammatory medication (including non-steroidal anti-inflammatory drugs)
22. Known allergy to milk or soy
23. Any other reason the investigator felt the subject was not suitable for participation in the study

MRI – Magnetic resonance imaging

Supplementary Table S4. Schedule of study events

**Assessment**	**Intervention 1 (4 weeks)**					**Washout (4 weeks, w5-8)**	**Intervention 2 (4 weeks)**				
	Baseline 1 w0	w1	w2	w3	w4		Baseline 2 w8	w9	w10	w11	w12
**Resting state fMRI**					**x**						**x**
**Structural MRI**					**x**						**x**
**Morning saliva collection**	**x**				**x**		**x**				**x**
**Blood sampling**	**x**				**x**		**x**				**x**
**Daily questionnaires**	**x**						**x**				
**Weekly questionnaires**	**x**	**x**	**x**	**x**	**x**		**x**	**x**	**x**	**x**	**x**
**ANS measurement**	**x**				**x**		**x**				**x**
**Actigraphy**	**x**				**x**		**x**				**x**
**CO_2 test**	**x**										

w – Week, (f)MRI – (Functional) magnetic resonance imaging, ANS – Autonomic nervous system

Residuals for questionnaire analysis were checked for approximate normality by visual inspection

PSS:

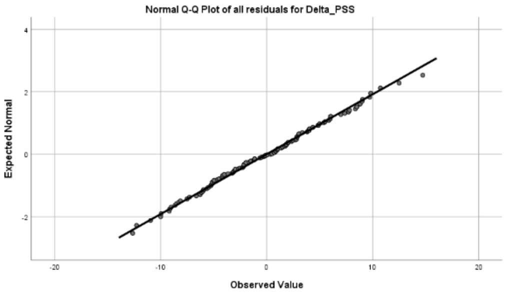

STATE:

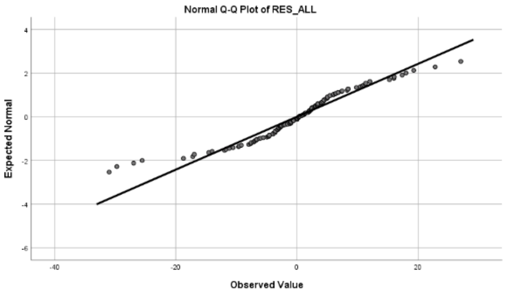

Euro-QoL:

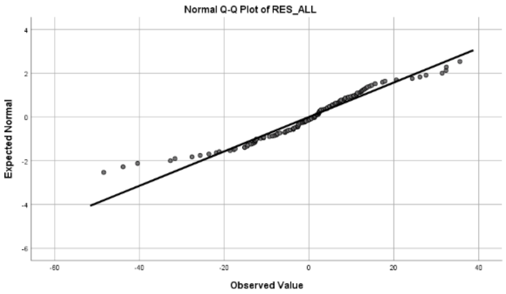

TRAIT:

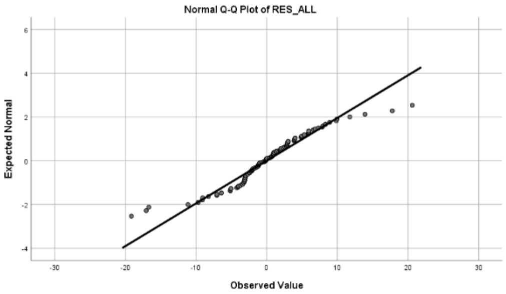

HADS_D:

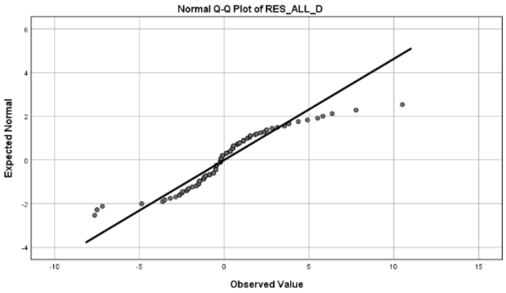

DOW:

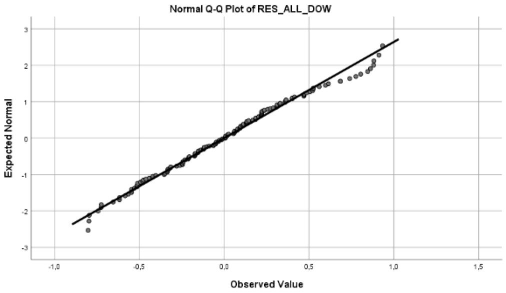

HADS_A:

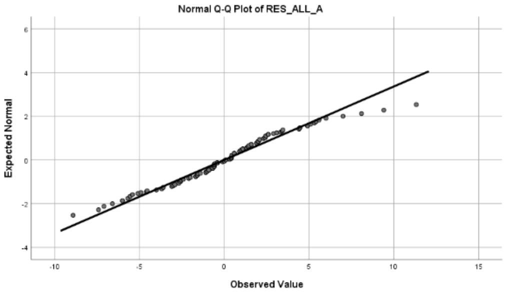

DOW health:

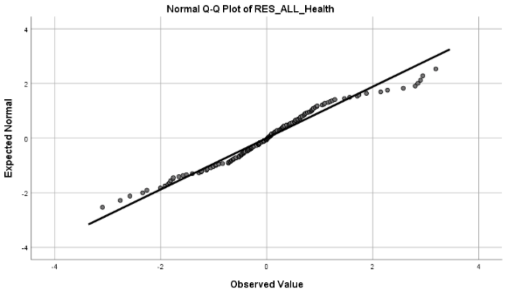

HADS_T:

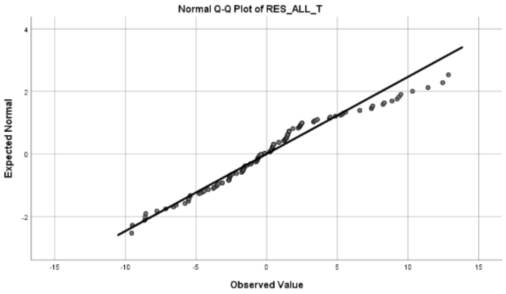

The CO_2 Challenge test

CO_2 is traditionally used in stress-inducing paradigms for anxiety, panic and stress disorders. The CO_2 challenge test consists of three double vital capacity inhalations, whereof the first and the last contain normal air, and the middle one a CO_2 gas mixture. In the present study, a gas mixture of 35% CO_2 and 65% oxygen was delivered using a nasal-oral face mask and a double vital capacity breath. During the test, minimum, maximum and average heart rate was monitored. The participants rated their current level of fear or discomfort on visual analogous scales (VAS 1-100) in multiple instant assessments for the duration of 60 s. Additionally, the Panic Symptom List (PSL-IV) was used to evaluate panic symptomatology. It consists of thirteen items, each item representing a DSM-IV panic symptom, to be rated on a five-point scale, from 0 (absent) to 4 (very intense). For the classification of responders and non-responders an increase of at least one point in four different PSL symptoms [1] or an increase of the average VAS rating of more than the mean + 2 SD before CO_2 inhalation [2] was used.

Statistical analysis

For the CO_2 test, the total score of the PSL and the average, minimum and maximum score as well as AUC of the VAS, and the minimum, maximum and average heart rate were calculated. Normal distribution was assessed by the Shapiro-Wilk test. As data were non-normally distributed, differences in reactions towards the inhalations were analysed using a Friedman test with post hoc Dunn's comparison using GraphPad Prism version 8. Due to technical issues, heart rate monitoring was not available for one participant.

CO_2 inhalation evoked anxiety symptoms (baseline parameter)

Inhalation of 35% of CO_2 caused panic, anxiety and discomfort symptoms according to PSL and VAS (Supplementary Table 5). The CO_2 inhalation resulted in significantly increased total PSL, average and maximum VAS ratings as well as VAS AUC ($p<0.001$) compared to all other inhalations and control conditions. Only the minimum VAS rating did not differ significantly between the inhalations/conditions. Maximum, but not minimum nor average heart rate was significantly ($p=0.017$) higher during CO_2 inhalation compared to the practice inhalation at start, but not compared to the two experimental air inhalations. 4 participants out of 22 were identified as non-responders by total PSL and average VAS scores during CO_2 inhalation compared to before. 2 additional participants rated their symptoms just at the limit for distinction of responders and non-responders.

Supplementary Table S5. CO_2 inhalation test. The inhalation of 35% CO_2 resulted in significantly increased total PSL and VAS ratings in experiment 2 compared to all other inhalations and control conditions. Heart rate was not majorly affected. Only maximum heart rate differed between the inhalation in experiment 2 and practice. All other conditions/inhalations did not differ significantly. Multiple VAS was solely collected for experiment 1-3, thus average, minimum and maximum heart rate are the same for the other conditions, and AUC could not be calculated for those. Heart rate was solely collected during the inhalations (practice and experiment 1-3). Median and interquartile range (25 to 75) are presented. * p<0.05, ** p<0.001

	**10 minutes before**	**Practice**	**Experiment 1 (air)**	**Experiment 2 (CO_2)**	**Experiment 3 (air)**	**10 minutes after**
**Total PSL**	1.0 (0.8 to 2.3) **	1.0 (0.0 to 2.3) **	1.0 (0.0 to 3.0) **	18.0 (11.3 to 23.3)	1.0 (0.0 to 3.3) **	0.0 (0.0 to 1.3) **
**Average VAS 0-100**	8.0 (0.0 to 12.8) **	4.0 (1.0 to 12.0) **	3.5 (1.0 to 7.3) **	51.0 (37.5 to 64.0)	2.5 (1.0 to 8.3) **	1.0 (0.0 to 4.0) **
**Minimum VAS 0-100**	8.0 (0.0 to 12.8)	4.0 (1.0 to 12.0)	1.0 (0.0 to 4.3)	4.0 (0.0 to 9.3)	0.3 (0.0 to 4.3)	1.0 (0.0 to 4.0)
**Maximum VAS 0-100**	8.0 (0.0 to 12.8) **	4.0 (1.0 to 12.0) **	7.5 (2.8 to 10.3) **	71.0 (55.8 to 85.3)	8.3 (2.8 to 15.5) **	1.0 (0.0 to 4.0) *
**AUC VAS 0-100**	nd	nd	221.5 (37.5 to 431.5) **	2258 (1432 to 3067)	148.0 (40.75 to 508.8) **	nd
**Average heart rate**	nd	105.0 (66.5 to 118.5)	101.0 (79.0 to 139.0)	109.0 (82.0 to 127.0)	105.0 (76.0 to 145.0)	nd
**Minimum heart rate**	nd	65.0 (56.5 to 80.5)	73.0 (60.5 to 91.5)	63.0 (43.0 to 89.0)	68.0 (54.5 to 78.0)	nd
**Maximum heart rate**	nd	159.0 (83.5 to 184.5) *	169.0 (105.0 to 196.5)	180.0 (101.0 to 206.5)	181.0 (100.0 to 203.0)	nd

CO_2 – carbon dioxide, PSL – Panic symptom list, VAS – Visual analogous scale, AUC – Area under the curve

References

1. Poma SZ, Milleri S, Squassante L, et al. Characterization of a 7% carbon dioxide (CO2) inhalation paradigm to evoke anxiety symptoms in healthy subjects. J Psychopharmacol 2005;19:494-503.
2. Griez EJ, Colasanti A, van Diest R, et al. Carbon dioxide inhalation induces dose-dependent and age-related negative affectivity. PLoS One 2007;2:e987.
