# Supplementary figures and images for: Multi-Strain Probiotic Mixture Affects Brain Morphology and Resting State Brain Function in Healthy Subjects: An RCT
Source: Cells. 2022 Sep 19;11(18):2922. doi: 10.3390/cells11182922 (PMC9496704; doi:10.3390/cells11182922)

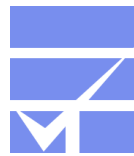

# CONSORT

TRANSPARENT REPORTING of TRIALS

## CONSORT 2010 Flow Diagram

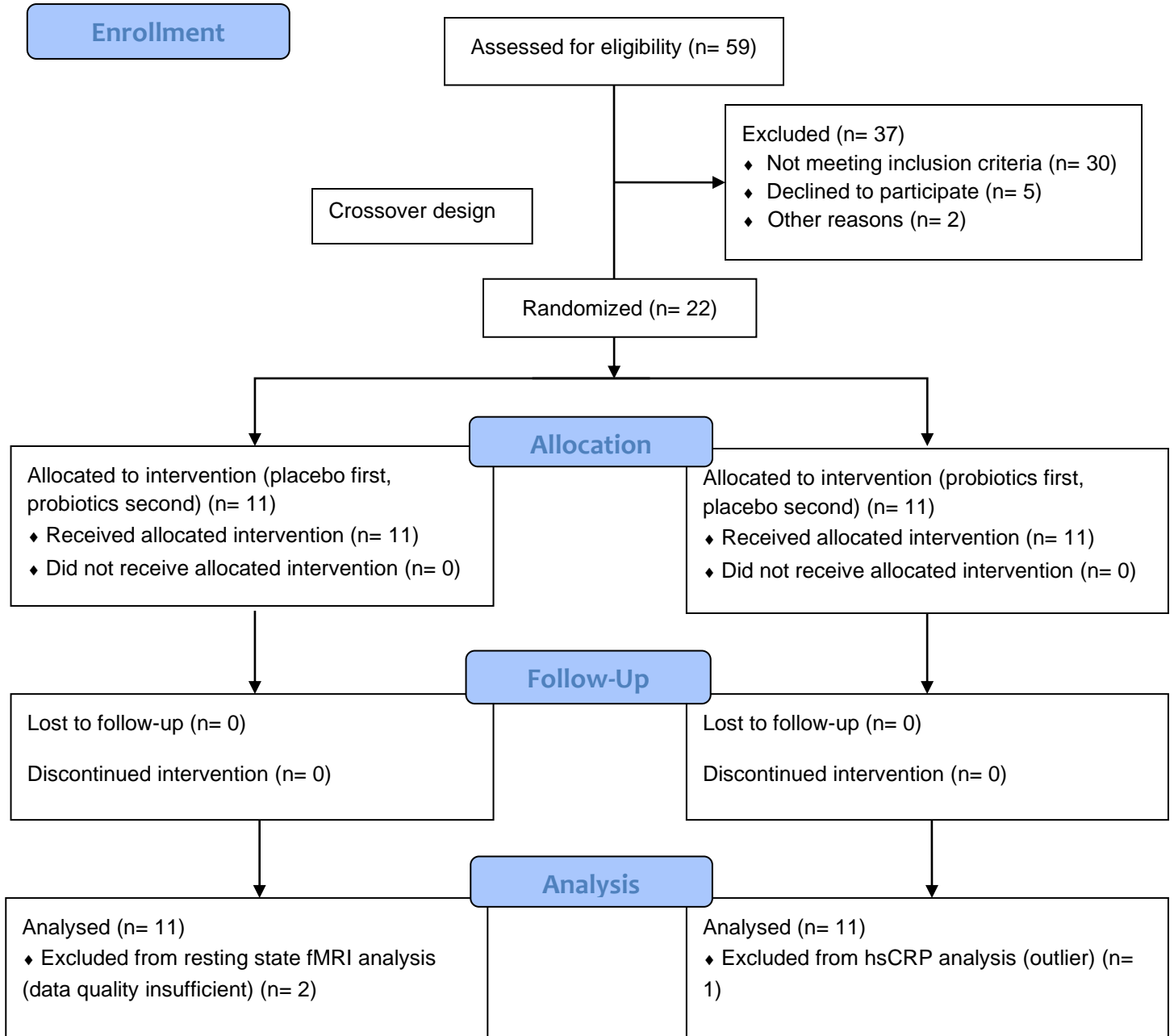

Supplement: Supplementary file 1 [file cells-11-02922-s001.zip › PROBIO_VBM+REST_CONSORT 2010 Flow Diagram.pdf]
